# Content analysis of health-related subjects in the K12 school curricula of Japan, Indonesia, Philippines, Guam, Micronesia, Marshall Islands, Palau, and Fiji

**DOI:** 10.1186/s41182-023-00511-1

**Published:** 2023-03-30

**Authors:** Akihiro Nishio, Fumiko Shibuya, Calvin S. de los Reyes, Crystal Amiel M. Estrada, Ernesto R. Gregorio, Dian Puspita Sari, Cut Warnaini, Hamsu Kadriyan, Maria Sandra M. Cruz, Margaret Hattori-Uchima, Paul Dacanay, Rudelyn Dacanay, Hillia Langrine Enos, Tarmau Terry Ngirmang, Mohamed Khalif, Saula Golea Volavola, Sachi Tomokawa, Mika Kigawa, Jun Kobayashi

**Affiliations:** 1 Health Administration Center, Gifu University, Gifu, Japan; 2 Department of Global Health, Graduate School of Health Sciences, University of the Ryukyus, Sembaru, Japan; 3 Japanese Consortium for Global School Health Research, Sembaru, Japan; 4 Department of Behavioral Sciences, College of Arts and Sciences, University of the Philippines Manila, Manila, Philippines; 5 Departments of Environmental and Occupational Health, College of Public Health, University of the Philippines Manila, Manila, Philippines; 6 SEAMEO TROPMED Regional Centre for Public Health, Mataram, Philippines; 7 Deparment of Health, Promotion and Education, College of Public Health, University of the Philippines Manila, Manila, Philippines; 8 Faculty of Medicine, University of Mataram, Pohnpei, Indonesia; 9 School of Health, University of Guam, Mangilao, USA; 10 College of Micronesia-FSM, Pohnpei, Federated States of Micronesia; 11 Ministry of Health and Human Service of the Marshall Islands, Majuro, Marshall Islands; 12 Palau Community College, Koror, Palau; 13 Ministry of Education, Heritage and Arts of Fiji, Suva, Fiji; 14 World Health Organization Representative Office for the South Pacific, Suva, Fiji; 15 Faculty of Education, Shinshu University, Nagano, Japan; 16 Faculty of Health and Social Services, Kanagawa University of Human Services, Yokosuka, Japan

**Keywords:** Health education, Curricula, School, Hygiene, Mental health, Nutrition, Education for sustainable development (ESD)

## Abstract

**Background:**

As a component of health promoting school, a school curriculum for health education was considered a fundamental. This survey aimed to identify the components of health-related topics and in which subjects were they taught.

**Methods:**

Four topics were chosen: (i) hygiene, (ii) mental health, (iii) nutrition-oral Health, and (iv) environmental education related to global warming in Education for Sustainable Development (ESD). Before gathering the curricula from partner countries, school health specialists were gathered to discuss the appropriate components of a curriculum that required evaluation. The survey sheet was distributed to and answered by our partner in each country.

**Results:**

About hygiene, individual practices or items that improve health-related were widely covered. However, items that imparted health-related education from an environmental perspective were not widely covered. About mental health, two types of country groups were identified. The first group included countries that taught mental health topics mainly as part of morals or religion; the second group included countries that imparted mental health topics mainly as part of health. The first group focused mainly on communication skills or coping methods. The second group focused not only on communication and coping skill but also on basic knowledge of mental health. About nutrition-oral education, three types of country groups were identified. One group imparted nutrition-oral education mainly in terms of health or nutrition. Another group imparted this topic mainly in terms of morals, home economics, and social science. The third group was the intermediate group. About ESD, a solid structure for this topic was not identified in any country. Many items were taught as part of science, while some were taught as part of social studies. Climate change was the most commonly taught item across all countries. The items related to environment were relatively limited compared to those related to natural disasters.

**Conclusions:**

Overall, two different approaches were identified: the cultural-based approach, which promotes healthy behaviors as moral codes or community-friendly behaviors and the science-based approach, which promotes children’s health through scientific perspectives. Policymakers should initially  consider the findings of this study while making decisions on which approach should be taken.

## Background

Children and adolescents spend most of their time in schools. Thus, promoting healthy behavior from early childhood through the school setting can directly benefit children. The World Health Organization (WHO) defines a health promoting school as “a school that is constantly strengthening its capacity as a healthy setting for living, learning and working.” Health promoting schools (HPS) have been recognized as strategic vehicles to not only promote positive development and healthy behaviors such as physical activity, physical fitness, recreation, play, and balanced nutrition but also prevent tobacco use, bullying, and aggressive behavior [1]. The systematic survey assessed the impacts of HPS among low- and middle-income countries in Western Pacific Region and stated that all eight identified studies showed significant knowledge and attitudes changes [2]. Nowadays, HPS is considered a fundamental value to promote children’s health. To enhance HPS, the WHO launched eight Global Standards on Health Promoting Schools in 2021 [3]. These eight global standards aim at the progressive realization of HPS and cover (i) government policies and resources, (ii) school policies and resources, (iii) school governance and leadership, (iv) school and community partnerships, (v) school curriculum, (vi) school social environment, (vii) school physical environment, and (viii) school health services. Among these eight standards, a school curriculum for health education was considered a fundamental part of an overall school health program. It provides young people with the knowledge and skills they need to become successful learners and healthy and productive adults. Choosing or developing a well-structured health education curriculum which covers widely essentials components is a critical step in ensuring that health education effectively promotes healthy behaviors. The curriculum selection or development process, however, can lack structure and focus, which can result in choosing or developing curricula that are inadequate or ineffective.

EDU-Port Japan, supported by the Ministry of Education, Culture, Sports, Science and Technology, Japan, is a “public–private, nationwide” initiative to provide a platform in which the public and private sectors collaborate to achieve global education projects. The “Research Project for promoting Japanese-style Education Amidst the COVID-19 Pandemic (EDU-Port Japan 2.0)” began in 2021. The EDU-Port Japan project, organized by the University of the Ryukyus, held an online consultation workshop on healthy and safe schools for the post-COVID-19 era in the Asia–Pacific Islands on January 26, 2022, in cooperation with WHO, Western Pacific Regional Office. The University of the Ryukyus had a partnership with the University of the Philippines-Manila, Mataram University from Indonesia, and the University of Guam. This survey was a part of multiple surveys of the Eduport projects. Other surveys focused on COVID-19 managements at schools, and several countries reports about school health. This study aims to provide basic information on school health among the targeted countries.

The COVID-19 pandemic has had a major impact on children's health and education in the Asia–Pacific region after the SARS-CoV-2 delta variants began spreading globally. This has resulted in an increased risk of infection in schools and adverse mental health among children due to abuse not only at home but also online as a result of the prolonged school closures and the economic impact on families and communities. In addition, the risk of lifestyle-related diseases has also increased due to decreased physical activity. The importance of education regarding proper mental health and nutrition may be reconfirmed among educational and health experts in the region [4]. The increase in the incidence of emerging infectious diseases may be due, in part, to the rapid expansion of human settlements in recent years, which has resulted in greater instances of human–wildlife conflict. Because this is also related to the issue of global warming, it is necessary to systematically study the relationship between the environment and health [5]. Therefore, in this study, we will focus on hygiene, mental health, nutrition-oral health, and environmental education related to global warming. Since the curricula are not so flexible to be changed during the pandemic. Thus, this survey is considered as a baseline survey. By identifying the similarities and differences in the content of the curricula, we hope to make recommendations on how to improve the curriculum in each country.

## Methods

### Development of questionnaires

Before gathering the curricula from partner countries, school health specialists were gathered to discuss the appropriate components of a curriculum that required evaluation. All five specialists belonged to the Japanese school health consortium and worked in their own field for school health implementation. The following four topics were chosen: (i) hygiene, (ii) mental health, (iii) nutrition-oral Health, and (iv) environmental education related to global warming in Education for Sustainable Development (ESD). To develop the items of the curriculum survey, global guidelines were considered, if available. Other items were added by the specialists. Since we could not find a global standard of mental health curriculum, the “Guideline of African School Mental Health Curriculum” [6] and “Mental Health and High School Curriculum Guide (Version 3)” [7] were referred to as standards of the mental health curriculum. The agenda of “UN food Systems Summit in 2021” [8] and the article of “Health-promoting schools: an opportunity for oral health promotion” which was published in Bulletin of the World Health Organization [9] were used as global standards of the nutrition-oral health curriculum. A global standard of hygiene education could not be found. Since ESD is a concept that can be integrated into any subject, only components related to natural disasters and the environment were selected for this survey. The items were modified each time after initial meetings with partner countries. The subjects that contained these items were determined for primary, secondary, and high school curricula. The survey sheet was distributed to and answered by our partners in each country. Online meetings were repeatedly held and some items were revised in this step.

Since the names of subjects varied, they were classified into the following large categories: language (foreign language and local language), science, social studies, health, morals, religion, nutrition, home economics, physical education (PE), and integrated study. The definition of each item was elaborated to be understood clearly by participants during the survey. Table 1 depicts the final version of the items of this survey that was conducted from October 2021 to October 2022. This survey analyzed the published curricula of the countries. Table 2 shows the data sources of curricula of each country. The approval of ethical committee was not required.

**Table 1:** Items of the school health curriculum survey

Hygiene	Mental health	Nutrition-oral health	Education for sustainable development (ESD)
Handwashing with soap	Basic understanding of mental health or mental illness (described mentally good/bad status and reasons that lead to bad mental status)	Safety of foods	Climate change/global warming
Maintaining physical cleanliness (shower, haircut, etc)	More specific knowledge of mental illness (mental illness are clearly mentioned and explained)	Food supply system	A rise in sea level
Maintaining cleanliness of clothes	Understanding mental developmental stage (basic knowledge of mental developmental stage of human being. Just development of body is excluded)	Healthy eating	Glacial retreat
Ensuring environmental hygiene (house, room, toilet, etc)	Mutual understanding among peers	No sugar	Heatwaves
Ventilation of rooms	Communication with family members (the relationship between health and family is clearly mentioned)	No alcohol	Droughts
Hygienic management of food and cooking utensils	Seeking help and finding support	No smoking	Flooding
Prevention of injury	Self-awareness (personality or sense of value is mentioned. Self assessment for educational goal is excluded)	Oral health education	Winter storms
First-aid treatment	Management of emotions (in the meaning of avoiding conflicts. Concentration on learning is excluded)	Basic cooking skills	Hurricanes
The concept of health and the causes of disease	Stress management and coping	Handling of cooking facilities, cooking utensils and tableware	Wildfires
Infectious diseases	Managing conflict	Food culture (relationship between local food, local society, and natural condition)	
Menstrual hygiene	Positive mental health (ways for boosting good mental health are clearly mentioned. Simple exercise or physical activity is excluded)	Respect to life of animals and plants	
Clean water	Stigma toward mental illness		
Garbage problem			
Pollution of the air, land, water, etc			

This table describes the items and their definition of the health-related components of curricula

**Table 2:** Data sources of curricula of each country

Country	Curriculum	Curriculum from online database
Japan	Ministry of education, culture, sports, science and technology—Japan curriculum	https://www.mext.go.jp/a_menu/shotou/new-cs/1384661.htm
Indonesia	Indonesian curriculum	https://www.futureschool.com/indonesia-curriculum/
Philippines	K to 12 Basic education curriculum	https://www.deped.gov.ph/k-to-12/about/k-to-12-basic-education-curriculum/
Guam	Curriculum and Instruction	http://guam.cyberschool.com/District/Department/5-Curriculum-Instruction/Portal/resource-page
Palau	Ministry of Education Syllabus	Not available online
FSM	Pnhnpei department of education curriculum frameworks 2009	https://national.doe.fm/index.php/ndoe-public/education-documents/education-curriculum-and-slo/496-science-curriculum
	National curriculum standards and benchmarks DOE 2008	https://national.doe.fm/index.php/ndoe-public/education-documents/education-curriculum-and-slo/
	National curriculum standards and benchmarks DOE 2008 (CHUUK EDUCATION REFORM)	https://national.doe.fm/PublicDocuments/Education%20Strategic%20Plans/ChuukDOE-Reform-Plan-2011-to-2015-Final.pdf
Marshall Islands	MOE Curriculum (K-12)	http://katakcenter.org/moe_docs.html#
Fiji	The Fiji Islands National Curriculum Framework	http://www.paddle.usp.ac.fj/collect/paddle/index/assoc/fj33.dir/doc.pdf
	Ministry of Education Heritage and Arts	https://www.futureschool.com/fiji-curriculum/#top

This table shows the online sources of curricula of each country. The online source was not available only in Palau

### Targeted countries

Initially, the curricula of Japan, Indonesia, the Philippines, and Guam were analyzed in 2021. The result was showen in the online consultation workshop “Healthy and Safe Schools for the Post-COVID-19 Era in the Asia–Pacific Islands” which was held on January 26, 2022. The invitation letters for this workshop were sent to all Western Pacific Islands. The further expansion of the targeted countries was discussed at the workshop. Finally, the participants who belonged to the Federated States of Micronesia (FSM), Marshall Islands, Palau, and Fiji agreed to join in our survey. Thus, this survey was conducted for Japan, Indonesia, Philippines, and Guam in 2021 and for Micronesia, Marshall Islands, Palau, and Fiji in 2022.

## Results

### Hygiene

Overall, individual practices or items that improve health-related were widely covered. However, items that imparted health-related education from an environmental perspective were not widely covered.

The results are depicted in Table 3. Only FSM did not have a hygiene-related curriculum. Palau’s hygiene-related curriculum comprised just one page and was thus, very limited. Apart from these two countries, hygiene-related curricula were widely covered in primary school. In Fiji, hygiene-related topics were taught as part of moral studies in early primary school before transitioning to topics related to health in later primary school. Hygiene-related topics were more likely to be taught in primary schools than in high schools in all countries. Individual practices such as “handwashing with soap,” “maintaining physical cleanliness,” and “maintaining the cleanliness of clothes” or items that improve health-related knowledge such as “prevention of injury,” “first-aid treatment,” “the concept of health and the causes of disease,” and “infectious diseases” were widely covered in all countries, except FSM and Palau. However, items that imparted health-related education from an environmental perspective such as “clean water,” “garbage problem,” and “pollution of the air, land, water, etc.” were not widely covered.

**Table 3:** Hygiene-related items in the curricula

Target	Subcategory	Items of curricula	Japan	Indonesia	Philippines	Guam	FSM	Marshal Islands	Palau	Fiji
Primary School	Individual practices	Handwashing with soap	Health	Language	Health	Health		Health		Health
				Social studies						
		Maintaining physical cleanliness (shower, haircut, etc.)	Health	Health and PE	Health	Health		Health		Health
				Religion (Is, Ca, Ko)	Home economics					
				Language						
		Maintaining cleanliness of clothes	Home economics	Health and PE	Home economics	Health		Health		Health
					Health					
	Housing	Keeping environmental hygiene (house, room, toilet, etc.)	Science	Religion (Is, Ca)	Health			Social studies		Moral
			Home economics	Health and PE						
				Language	Home economics					Health
				Social studies						
		Ventilation of rooms	Home economics							
		Hygienic management of food and cooking utensils	Home economics		Home economics					
	Knowledge of hygiene	Prevention of injury	Science	Health and PE	Health	Health		Health	Health	Morals
										Health
		First-aid treatment	Health	Health and PE	Health	Health		Health		Health
		The concept of health and the causes of disease	Science	Language	Health			Health	Health	Morals
			Health	Science						Health
		Infectious diseases	Health	Health and PE	Health	Health		Health		Health
								Science		
		Menstrual hygiene		Health and PE	Health					Health
	Environment	Clean water			Health			Science		Morals
										Health
		Garbage problem		Language	Health					Morals
				Social Studies						Health
		Pollution of the air, land, water, etc			Health	Science		Science		Health
					Home economics					
Middle School	Individual practice	Handwashing with soap				Home economics				Health
						Health				
		Maintaining physical cleanliness (shower, haircut, etc.)		Religion (Is, Bu)		Health			Health	Health
		Maintaining cleanliness of clothes	Home economics	Religion (Is, Bu)		Health		Health		
	Housing	Keeping environmental hygiene (house, room, toilet, etc.)	Home economics	Social studies				Health		Health
		Ventilation of rooms								Health
		Hygienic management of food and cooking utensils	Home economics			Home economics		Nutrition		
	Knowledge of hygiene	Prevention of injury	Home economics	Health and PE	Health				Health	Health
			Health		Home economics					
		First-aid treatment	Health	Health and PE	Health					Health
					Home economics					
		The concept of health and the causes of disease	Health	Health and PE	Health			Health	Health	Health
		Infectious diseases	Health		Health			Health		Health
		Menstrual hygiene								Health
	Environment	Clean water			Health			Health		Health
		Garbage problem			Health					Health
		Pollution of the air, land, water, etc			Health			Health		Health
								Science		
High School	Individual practice	Handwashing with soap		Health and PE		Home economics				
						Health				
		Maintaining physical cleanliness (shower, haircut, etc.)		Health and PE		Health		Nutrition		
		Maintaining cleanliness of clothes	Home economics							
	Housing	Keeping environmental hygiene (house, room, toilet, etc.)	Home economics	Health and PE				Health		
		Ventilation of rooms								
		Hygienic management of food and cooking utensils	Home economics		Home economics	Home economics				
			Health							
	Knowledge of hygiene	Prevention of injury		Health and PE	PE	Health				
		First-aid treatment	Health		Home economics			Health		
		The concept of health and the causes of disease	Health	Health and PE				Health		
		Infectious diseases	Science	Science		Health		Health		
			Health							
		Menstrual hygiene								
	Environment	Clean water								
		Garbage problem		Science	Home economics					
		Pollution of the air, land, water, etc		Science						
				Social studies						

FSM did not have any curriculum which educated hygiene-related items. Individual practices and health-related knowledge were widely covered in many countries. However, environmental items were not widely covered

*Is*  Islam, *Ca*  Catholicism, *Cr*  Christianity, *Hi*  Hinduism, *Ko* Confucianism, *Bu*  Buddhism

### Mental health

The results are depicted in Table 4. Two types of country groups were identified. The first group included countries that taught mental health topics mainly as part of morals or religion; the second group included countries that imparted mental health topics mainly as part of health. Japan and Indonesia belonged to the first group, while the remaining countries belonged to the second group. Only FSM did not have a subject that widely covered mental health-related topics. The first group focused mainly on communication skills such as “mutual understanding among peers,” “communication with family members,” “seeking help and finding support,” and “managing conflict” or coping methods such as “self-awareness,” “management of emotions,” “stress management and coping,” and “positive mental health.” The second group focused not only on communication and coping skill but also on basic knowledge of mental health such as “basic understanding of mental health or mental illness” and “understanding of mental developmental stage.” Although the stigma toward mental illness may be one important component, this aspect was not independently elaborated by any country. “More specific knowledge of mental illness” was imparted only to middle-school students in the Philippines. In Indonesia, some items were taught as part of the language. Moreover, the timing and components of mental health-related items depended on students’ religions. In the Philippines, mental health-related items shifted from health subjects in primary and middle schools to moral subjects in high schools.

**Table 4:** Mental health-related items in the curricula

Target	Subcategory	Items of curricula	Japan	Indonesia	Philippines	Guam	FSM	Marshall Islands	Palau	Fiji
Primary school	Knowledge of mental health	Basic understanding of mental health or mental illness			Health	Health		Health		
		More specific knowledge of mental illness								
		Understanding mental developmental stage		Religion (Ch)	Health					Health
				Science	Home economics					
	Communication skill	Mutual understanding with peers	Integrated studies	Religion (Is, Ch, Ca, Hi)	Health	Health		Language	Health	Morals
				Social studies						
			Morals	Language		Social studies			PE	Health
		Communication with family members	Morals	Religion (Is, Hi, Ko)	Health	Health			Health	Morals
				Language		Social studies				Health
				Social studies						
		Seeking help and finding support			Health	Health		Health	Health	Morals
						Social studies				Health
	Coping method	Self-awareness	Integrated studies	Religion (Is, Ch, Ca)	Health	Health				
		Management of emotions	Integrated studies	Religion (Is)	Health	Health				Health
		Stress management and coping	Integrated studies							Health
						Health				
		Managing conflict	Integrated studies	Religion (Is, Ch)	Health	Social studies	Social studies	Social studies	Health	Health
		Positive mental health	Integrated studies		Health	Health				Health
	Other	Stigma toward mental illness								
Middle school	Knowledge of mental health	Basic understanding of mental health or mental illness			Health	Health		Health		Health
		More specific knowledge of mental illness			Health					
		Understanding developmental stage	Integrated studies		Health					Health
	Communication skill	Mutual understanding with peers	Integrated studies	Religion (Is, Ch, Hi)		Health		Language	Health	Health
			Morals					Language		
		Communication with family members	Morals	Social studies		Health		Health	Health	Health
		Seeking help and finding support				Health				Health
	Coping method	Self-awareness	Integrated studies	Social studies	Health	Health		Language	Health	Health
				Religion (Is, Ch, Hi, Bu)						
		Management of emotions			Morals	Health				Health
		Stress management and coping			Health			Health		Health
		Managing conflict					Social studies	Health		Health
								Social studies		
		Positive mental health								
	Other	Stigma toward mental illness								
		Other (violence, bullying)		Health and PE	Morals					Health
		Other (child protection)			Morals					
High school	Knowledge of mental health	Basic understanding of mental health or mental illness			Morals	Health				
		More specific knowledge of mental illness								
		Understanding developmental stage		Religion (Ch)	Morals	Home economics				
	Communication skill	Mutual understanding with peers	Integrated studies	Religion (Ch)	Morals	Health				
		Communication with family members		Religion (Ch, Is)	Morals	Health		Health		
		Seeking help and finding support				Health				
	Coping method	Self-awareness	Integrated studies	Religion (Is, Ch)	Morals	Health				
		Management of emotions		Religion (Ch)	Morals	Health				
		Stress management and coping			Morals	Health				
		Managing conflict		Religion (Ch)		Health	Social Studies	Social studies		
								Health		
		Positive mental health			Morals	Health				
	Other	Stigma toward mental illness								
		Other (suicide prevention)		Religion (Ca)						

Japan and Indonesia taught mental health topics mainly as part of morals or religion. Other countries imparted mental health topics mainly as part of health. Only FSM did not have a subject that widely covered mental health-related topics

*Is*  Islam, *Ca*  Catholicism, *Cr*  Christianity, *Hi*  Hinduism, *Ko* Confucianism, *Bu*  Buddhism

### Nutrition-oral education

The results are depicted in Table 5. The items related to nutrition-oral education were taught mainly as part of health, home economics, and social studies. “Healthy eating” was the most common item and was taught in all countries, except FSM. “Food culture” was also common and was taught mainly as part of health or social studies in all countries, except Indonesia and Palau. “Oral health education” was taught in all countries, except FSM and Palau. In general, this topic was more likely to be taught in primary schools than in high schools. Three types of country groups regarding nutrition-oral education were identified. One group, which included the Philippines, the Marshall Islands, and Fiji, imparted nutrition-oral education mainly in terms of health or nutrition. Another group, comprising Japan and FSM, imparted this topic mainly in terms of morals, home economics, and social science. The third group was the intermediate group comprising Indonesia, Guam, and Palau. “Healthy eating” was the most commonly discussed item, followed by “food culture.”

**Table 5:** Nutrition-oral education-related items in the curricula

Target		Items of curricula	Japan	Indonesia	Philippines	Guam	FSM	Marshall Islands	Palau	Fiji
Primary school	Food security	Safety of foods	Morals		Health					Health
			Home economics							
		Food supply	Social studies		Health		Social studies			Health
			Science		Home economics		Science			
	Healthy food	Healthy eating	Home economics	Health and PE	Health	Health		Health	Health	Moral
			Health and PE	Language	Home economics					Health
	Oral health	No sugar			Health			Health		
		No alcohol		Health and PE	Health			Health		
		No smoking		Health and PE	Health			Health		
		Oral health education	Health and PE	Language	Health	Health		Health		Morals
	Cooking skills	Basic cooking	Home economics		Health			Social studies		
		Handling of cooking facilities, cooking utensils and tableware	Home economics		Health					Health
	Food culture	Food culture	Science		Health	Health	Social studies	Social studies		Health
			Morals			Social studies				
			Home economics							
		Respect for food	Science	Social studies	Health					
			Morals							
	Other	Other (avoiding substances abuse)				Health				
Middle school	Food security	Safety of foods				Home economics				Health
		Food supply	Social studies			Home economics	Social studies	Social studies		Health
							Science			
	Healthy food	Healthy eating	Home economics	Science		Health		Nutrition	Health	Health
				Health and PE		Home economics				
	Oral health	No sugar								Health
		No alcohol			Health			Health		Health
		No smoking			Health					Health
		Oral health education	PE			Health				
	Cooking skills	Basic cooking	Home economics			Home economics		Nutrition		
								Social studies		
		Handling of cooking facilities, cooking utensils and tableware			Home economics	Home economics		Nutrition		
								Health		
	Food culture	Food culture	Social studies				Social studies	Social studies	Social studies	Health
			Home economics					Nutrition		
		Respect for food	Moral education							
	Other	Other (additive and addictive substances in food or beverages)		Science						
High school	Food security	Safety of foods			Home economics	Home economics		Nutrition		
		Food supply				Home economics	Social studies			
							Science			
	Healthy food	Healthy eating		Health and PE	Health and PE	Home economics		Health		
				Science		Health				
	Oral health	No sugar						Nutrition		
		No alcohol		Health and PE		Health		Nutrition		
		No smoking		Health and PE		Health				
				Science						
		Oral health education				Health		Nutrition		
	Cooking skills	Basic cooking				Home economics		Nutrition		
		Handling of cooking facilities, cooking utensils and tableware			Home economics	Home economics		Nutrition		
	Food culture	Food culture			Social studies		Social studies	Social studies		
		Respect for food		Science						
	Other	Other (substance abuse)				Health				

The items related to nutrition-oral education were taught mainly as part of health, home economics, and social studies. “Healthy eating” was the most common item and was taught in all countries, except FSM

### Environmental education related to global warming in education for sustainable development (ESD)

The results are depicted in Table 6. “Climate change” was the most common item and was taught to middle-school students in all countries mainly as part of science or social studies. Fiji taught all of the contents of ESD as part of health. The items related to the environment were relatively limited compared to those related to natural disasters. As with other topics, some of the items were taught as part of language in Indonesia.

**Table 6:** ESD-related items in the curricula

Target	Subcategory	Items of curriculum	Japan	Indonesia	Philippines	Guam	FSM	Marshall island	Palau	Fiji
Primary school	Climate change	Climate change/global warming		Language		Science	Science	Social studies	Science	
							Social studies			
		A rise in sea level				Science		Social studies	Science	
		Glacial retreat				Science				
	Natural disaster	Heatwaves				Science				
		Droughts		Language		Science				
		Flooding	Social studies		Health	Science				
		Winter storms				Science				
		Hurricanes	Social studies			Science			Science	
			Science							
		Wildfires				Science				
		Other (natural disaster in general)		Language					Science	
		Other (emergency-related drills and training)								Health
	Environment	Other (preservation of natural resources)		Science						
		Other (ecosystem)		Science						
		Other (recycling)			Home economics		Science			Health
		Other (pollution)					Social studies			
		Other (resource protection)								
Middle school	Climate change	Climate change/global warming	Science	Science	Health	Science	Science	Science	Science	Health
			Home economics				Social studies	Social studies		
			Integrated studies							
		A rise in sea level				Science	Science	Science	Science	Health
		Glacial retreat				Science				
	Natural disaster	Heatwaves				Science				
		Droughts				Science	Science	Science		Health
		Flooding				Science		Science		
		Winter storms				Science				
		Hurricanes				Science	Science	Science		
		Wildfires				Science		Social studies		
		Other (natural disasters in general)		Science						
		Other (emergency-related drills and training)			Home economics					Health
	Environment	Other (pollution)		Science			Social studies			
		Other (the impact of technology on environment)		Science						
		Other (the impact of population growth)		Science						
		Other (existing global health initiatives)			Health					
		Other (recycling)					Science	Home economics		Health
High school	Climate change	Climate change/global warming	Social studies	Social studies	Science			Science		
			Science	Science				Social studies		
			Home economics							
		A rise in sea level						Science		
		Glacial retreat	Science							
	Natural disaster	Heatwaves			Science					
		Droughts		Social studies	Science					
		Flooding	Social studies	Social studies	Science					
		Winter storms								
		Hurricanes	Social studies							
		Wildfires		Social studies						
		Other (emergency-related drills and training)						Health		
		Other (mitigation and adaptation to natural disaster)		Social studies	Home economics					
	Environment	Other (food security and alternative source of energy)		Social studies						
		Other (environmental protection)		Social studies	Home economics					

“Climate change” was the most common item and was taught to middle-school students in all countries mainly as part of science or social studies

## Discussion

Overall, basic components that enhanced individual hygienic practices and knowledge of hygiene were generally taught in partner countries, except FSM and Palau. However, environmental-related items such as “clean water,” “garbage problem,” and “pollution of the air, land, etc.” were not widely taught. Hygienic actions being promoted at schools are shifting from individual behaviors to environmental activities these days [10–12]. The hygiene-related topics may be elaborated again from the environmental perspective.

About the subjects related to mental health, two trends were identified. One was that while some countries (Japan and Indonesia) taught this topic in terms of morals or religion (However, Japan introduced a new curriculum on mental health in 2022. Since this survey was conducted for Japan in 2021, the new curriculum was not assessed in this survey), others taught it in terms of health. The other trend was that communication skills were taught the most widely as mental health education in many countries. Whether the topic of mental health should be included as part of morals, religion, or health subjects may be noteworthy to establish a standardized mental health curriculum. This decision should be made before developing the curricula for the countries wanted to develop the curricula.

Regarding the subjects related to nutrition-oral education, three types of country groups were identified. One group imparted nutrition-oral education mainly in terms of health or nutrition. Another group imparted this topic mainly in terms of morals, home economics, and social science. The third group was the intermediate group. Although the Indonesian curriculum contained various health topics, “food culture” was not one of them. This might be because Indonesia has an enormous diversity of food culture. Indonesian food culture was shaped by the geographical characteristics of each region, various ethnicities, and religions, as well as the cultural assimilation that resulted from international trading and colonialism. Hence, every region has a different food culture depending on the natural, historical, and cultural uniqueness of the region [13]. The introduction of food culture in a national curriculum might be difficult due to the differences. Although other countries might have various cultures and ethnicities, only the Indonesian curriculum has lots of varieties depending on the religions to which students belonged. In addition, health-related topics were tried to educate children, associating with their culture. The match between individuals and their cultures confers benefits in many domains [14, 15]. People whose behavior aligns with their cultural norms tend to experience positive or pleasant feelings [16–18]. The decision regarding whether nutrition-oral education-related items would be taught as part of morals, home economics, social science, or health may help develop the curriculum of nutrition-oral education.

Since ESD remains a new topic in health education, a solid structure for this topic was not identified in any country. Many items were taught as part of science, while some were taught as part of social studies. Climate change was the most commonly taught item across all countries. The items related to the environment were relatively limited compared to those related to natural disasters. ESD was a new concept that was proposed at the World Summit on Sustainable Development in 2002, and it has been promoted internationally by UNESCO, as the lead agency, based on the “United Nations Decade of Education for Sustainable Development” framework [19] and the “UNESCO Global Action Program on Education for Sustainable Development (ESD)” [20] adopted at the 37th UNESCO General Conference in 2013. Despite the efforts of UNESCO or other agencies, subjects related to ESD were not well developed in the targeted countries. This is an issue that needs to be addressed in the future. Moreover, ESD is a comprehensive topic and does not deal only with global warming. Thus, introducing ESD into the curriculum while maintaining consistency with Sustainable Development Goals-related education, which is currently being introduced in several countries, should be considered.

Although we tried to find the surveys which compared the health-related curricula of several countries, any similar survey was not identified. However, we found an article that focused only on food education and compared the curricula of 11 countries (Czech Republic, Denmark, England, Iceland, Ireland, Japan, Norway, Scotland, Slovenia, Sweden) [21]. This study revealed that only Norway had a unique food curriculum, where food is the central topic. In all other countries, food is only one part of the curriculum which has different central learning, such as general health subjects, science, and so on. This study also mentioned that food education is included within mandatory, primary academic curriculums and addresses food literacy. This differs from existing literature that examines food education as an “add-on” to the curriculum and begins to address the lack of research about food education within curriculums. The result of this study matched our study. Even in high-income countries, food education was added to existing subjects in many countries.

In Indonesia, several health-related components are included in the Indonesian language subject. In our opinion, this seems a well-elaborated strategy to overcome the differences of religions and promote children’s health. This may offer insights to policymakers who want to develop their own curriculum based on their culture.

There may be two approaches to developing health-related curricula. One is the culture-based approach, followed by Japan and Indonesia. These countries attempt to promote healthy behaviors, enhancing them as moral codes or community-friendly behaviors. The other is a science-based approach, followed by the Philippines, the Marshall Islands, and Fiji. These countries attempt to promote children’s health through scientific perspectives. Guam may belong to the intermediate state. Relatively, FSM and Palau had not developed a health-related curriculum. The culture-based approach may have a bigger impact than the science-based approach in enhancing healthy behaviors among children. Moreover, it is easy to involve the community. The disadvantage of this approach is the complexity of its development and the difficulties of adapting to not only various ethnic/religious groups but also rapid social change. On the other hand, the advantage of the scientific approach is the ease of catching up with global trends and new academic findings and developing the curriculum. In our opinion, the culture-based approach may be attractive to policy-makers who want to retain their tradition. Thus, Japanese and Indonesian approaches should be further scrutinized in future research. As we know, there were not any similar studies that compared health-related curricula among certain countries. For this meaning, this survey might bring new insights to policy makers.

## Conclusion

The topic of hygiene was mainly taught based on individual practice or by enhancing knowledge of hygiene in several countries. The environmental perspective toward hygiene was not sufficiently imparted in some countries. Regarding the mental health topic, two trends were identified: while some imparted this topic mainly in terms of morals or religion, others imparted this topic in terms of health. Regarding nutrition-oral health, three groups were identified: one group imparted education on this topic in terms of health or nutrition; another in terms of morals, home economics, or social science; and the third was the intermediate group. ESD remains a new topic in health education, with no evidence of a solid structure in any country. Related knowledge was imparted as a part of science in many cases and social studies in some cases. Overall, two different approaches were identified: the cultural-based approach, which promotes healthy behaviors as moral codes or community-friendly behaviors and the science-based approach, which promotes children’s health through scientific perspectives. Policymakers should initially consider the findings of this study while making decisions on which approach should be taken.

### Limitation

This survey focused only on the national curricula to identify the basic structure of health education. Even if some topics were taught in moral or religion, it is not clear that they are taught not in scientific way. In addition, this survey was not aimed to reveal how deeply and widely the health-related topics were taught in the textbook level.

## Data Availability

OK.

## Abbreviations

ESD: Education for sustainable development
FSM: Federated States of Micronesia
HPS: Health promoting schools
PE: Physical education
WHO: World Health Organization
